# Multi-component chlorination intervention to reduce neonatal infections in healthcare facilities in western Kenya (CLEAN Trial): study protocol for a cluster randomized controlled trial

**DOI:** 10.64898/2026.09.18.26363445

**Published:** 2026-09-21

**Authors:** Yoshika S. Crider, Mwale Chiyenge, Erick Odoyo, Joyce Kisiangani, Jeremy Lowe, Josline Wangia, Blastus Bwire, Mwanzia Kioko, Kelly T. Alexander, Carol Nekesa, Lillian Musila, Phelgona Otieno, Amy J. Pickering

## Abstract

**Background:**

The proportion of births occurring at healthcare facilities is rising globally, yet the birthing environment in healthcare facilities in low-income settings is often contaminated with bacterial pathogens, including antibiotic-resistant pathogens, that can lead to serious infections for newborns and their mothers. There is a need for effective strategies to reduce environmental contamination in healthcare facilities to reduce infection risks among facility-born neonates and their mothers.

**Methods:**

We designed the CLEAN ( C h L orine to reduce E nteric and A ntibiotic resistant infections in N eonates) cluster randomized controlled trial in western Kenya to evaluate the impact of a multi-component chlorination intervention on environmental contamination and maternal and neonatal infection risks. Thirty-six medium-sized public health facilities will be randomized in a 1:1 allocation ratio to receive a passive chlorination technology for water supply treatment paired with a reliable supply of chlorine-based disinfectant or status quo. Up to 22,500 mothers-neonate dyads will be enrolled and followed from birth through 28 days to collect symptoms of infection and mortality, with a subset of mother-neonate dyads selected for rectal swab collection to measure rectal colonization with sepsis-associated bacterial species. Environmental samples will be collected to measure bacterial pathogens on staff hands, high-touch surfaces, and in water supply. The primary objectives of the study are to evaluate the impact of the intervention on the following outcomes: (1) rectal carriage of bacterial pathogens one week post-birth among facility-born neonates and their mothers, (2) cumulative incidence in the first 7 days post-birth of possible serious bacterial infection among facility-born neonates, and (3) cumulative incidence in the first 7 days post-birth of symptoms of possible maternal sepsis.

**Discussion:**

This study will generate evidence on the effectiveness of a novel chlorination intervention to reduce healthcare associated infections, including antibiotic resistant infections, and improve maternal and neonatal survival.

**Trial registration:**

Clinical Trials NCT06824350 . Registered 7 February 2025, https://clinicaltrials.gov/study/NCT06824350

## Full Text Availability

The license terms selected by the author(s) for this preprint version do not permit archiving in PMC. The full text is available from the preprint server.

